# Automatic MRI segmentation of para-pharyngeal fat pads using interactive visual feature space analysis for classification

**DOI:** 10.1186/s12880-017-0179-7

**Published:** 2017-02-14

**Authors:** Muhammad Laiq Ur Rahman Shahid, Teodora Chitiboi, Tetyana Ivanovska, Vladimir Molchanov, Henry Völzke, Lars Linsen

**Affiliations:** 1 Jacobs University, Bremen, Germany; 2 Fraunhofer MEVIS, Bremen, Germany; 3 Georg-August-Universität, Göttingen, Germany; 4 Ernst-Moritz-Arndt-Universität, Greifswald, Germany

**Keywords:** Para-pharyngeal fat pads segmentation, Upper airway segmentation, Interactive visual analysis tool, Obstructive sleep apnea (OSA), Magnetic resonance imaging (MRI)

## Abstract

**Background:**

Obstructive sleep apnea (OSA) is a public health problem. Detailed analysis of the para-pharyngeal fat pads can help us to understand the pathogenesis of OSA and may mediate the intervention of this sleeping disorder. A reliable and automatic para-pharyngeal fat pads segmentation technique plays a vital role in investigating larger data bases to identify the anatomic risk factors for the OSA.

**Methods:**

Our research aims to develop a context-based automatic segmentation algorithm to delineate the fat pads from magnetic resonance images in a population-based study. Our segmentation pipeline involves texture analysis, connected component analysis, object-based image analysis, and supervised classification using an interactive visual analysis tool to segregate fat pads from other structures automatically.

**Results:**

We developed a fully automatic segmentation technique that does not need any user interaction to extract fat pads. Our algorithm is fast enough that we can apply it to population-based epidemiological studies that provide a large amount of data. We evaluated our approach qualitatively on thirty datasets and quantitatively against the ground truths of ten datasets resulting in an average of approximately 78% detected volume fraction and a 79% Dice coefficient, which is within the range of the inter-observer variation of manual segmentation results.

**Conclusion:**

The suggested method produces sufficiently accurate results and has potential to be applied for the study of large data to understand the pathogenesis of the OSA syndrome.

## Background

Obstructive sleep apnea (OSA) is commonly associated with obesity [1] and is considered as a public health problem affecting, at least, 2–4% of the middle-aged population [2]. OSA is defined as a recurrent cessation of respiration during sleep associated with the obstruction of an upper airway [3]. Obesity is one of the known risk factors for OSA and is reported in at least 50% of adults with this syndrome [4]. Obesity is a special one among other common factors (e.g., gender, age, craniofacial features, pharyngeal abductor and dilator muscles) in that it is reversible and, therefore, may reduce OSA severity following weight loss [5].

The way by which obesity affects upper airway collapse during sleep is not completely understood and needs further studies. Either increased size of para-pharyngeal fat pads or reduced distance between left and right para-pharyngeal fat pads reduce pharyngeal airway volume [6]. A smaller pharyngeal airway space has been identified in patients with OSA syndrome [7] and may result from enlargement of surrounding soft tissues, small distance between left and right fat pads, altered craniofacial morphology or a combination of all [8]. It is clear, however, that upper airway anatomy is important in the pathogenesis of obstructive sleep apnea [9]. In order to fully understand upper airway anatomy, we need to examine the volume of the airway and surrounding upper airway structures [10]. Fat pads segmentation from MRI is of considerable importance in providing noninvasive information about them that helps radiologists to visualize and study their anatomy [11]. For these reasons, fat pads segmentation from head MRI is an essential part of epidemiological study of OSA, and success of the study mostly depends on the segmentation accuracy and automation.

There is a number of publications dealing with OSA pathogenesis, symptoms, and treatment, but only few publications are available discussing automatic segmentation of pharyngeal airway and surrounding soft tissues. The lack of automatic segmentation technique is a great limitation in analyzing large datasets and epidemiological cohort studies. Schwab et al. [7] manually performed volumetric analysis of upper airways to analyze anatomic alterations. Liu et al. [12] proposed a semi-automatic framework for upper airway segmentation using fuzzy connectedness. Here, an operator defined a volume of interest and seed points in T1- and T2-weighted MR images manually. Ivanovska et al. [11] presented a semi-automatic segmentation pipeline for pharyngeal air column. Their approach consists of three steps: smoothing, thresholding, and 2D and 3D connected component analysis. Whereas the first two steps are rather common, the third step provides a set of general rules for extraction of the pharyngeal airway. Their method takes less than one minute to delineate the pharyngeal airway. The approach needs a small amount of interaction like defining the ending axial slice of oropharynx. Shahid et al. [10] described a fully automatic segmentation technique to segregate pharyngeal airway from head MRI. They performed pre-processing, object-based image analysis (OBIA), visual analysis of the feature space, and a refinement and extraction of the pharyngeal air column. They utilized the concept of dividing the image into small groups called “*objects*” having same basic features at voxel level. In this way, they explored additional features of different regions at the object level to classify them using interactive visual analysis tool. Their approach does not need any user interaction and consumes less than half of a minute to produce the complete segmentation results.

In summary, over the last few years we noticed major advancements in the field of upper airway segmentation from MRI, prompted by an increased use of MRI for soft tissues. Although there has been an effort towards developing an automatic segmentation technique for upper airway analysis, segmentation algorithms for fat pads have not been developed yet.

## Methods

The first step towards the endeavor of an epidemiological cohort study is to develop a reliable automatic segmentation technique to segregate para-pharyngeal fat pads from the head MRI for the pathogenesis of OSA. In this paper, we describe a reliable automatic segmentation pipeline for the fat pads from MR images. In our segmentation methodology, we extract coarse objects as candidates, analyze their feature values using a multidimensional feature view, develop a supervised classifier using an interactive visual analysis tool, and finally refine the shape.

**Fig. 1:**
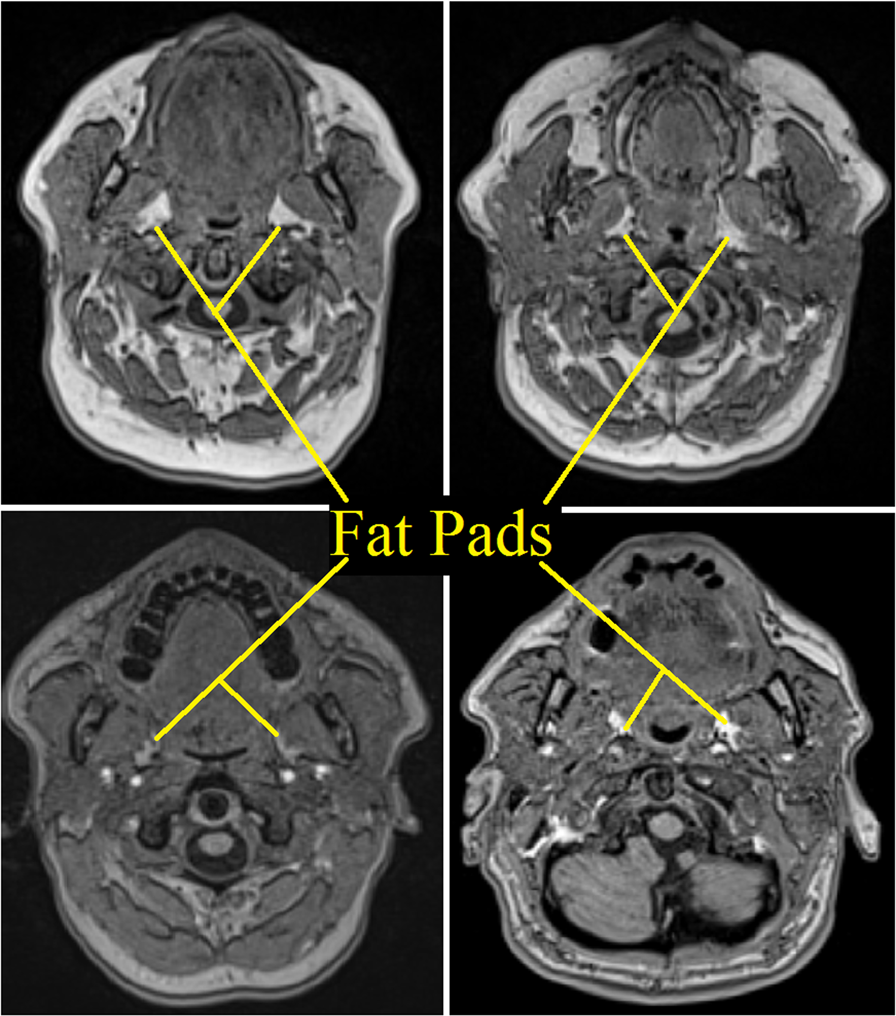
Diverse appearance of para-pharyngeal fat pads on axial slices

However, accurate and reproducible automatic segmentation of para-pharyngeal fat pads from head MRI meets many challenges. First of all, fat pad structures are inhomogeneous in terms of spatial repetitiveness of individual voxel intensities. They have irregular shape and diverse appearance in different subjects as shown in Fig. 1. They show inter- and even intra-subject variability of shapes, appearance, and textures. In some axial slices, fat pads are difficult to separate because of intensity values similar to blood vessels and other soft tissues. Consequently, a general segmentation algorithm may work well for one, but not for another subject, or only on certain axial slices of a particular subject.

Some of the challenges are due to the employment of MRI instead of computed tomography (CT) for airways imaging. Although, the X-ray CT is used more commonly for airways imaging and considered to be the gold standard for this purpose, its use in an epidemiological cohort study for research purposes is ethically not justified. Here, MRI as a non-radiation based scanning method gains an increasing popularity. MR imaging provides a good contrast between soft tissue structures and is used to study brain and throat tissues [13]. MRI makes it possible to conduct large-scale epidemiological cohort studies to analyze the role of fat pads in sleep apnea syndrome because it involves little or no risk to healthy volunteers. State-of-the-art segmentation methods for X-ray CT images are not applicable to the MRI, due to large differences in structure and tissue properties, and intensities.

Further challenges are imposed by the requirement of full automation of the segmentation technique. In practice, manual segmentation of fat pads is a tedious and time-consuming task. MRI scanners produce three dimensional images by generating multiple two-dimensional cross-sections (slices), and the radiologist or medical expert has to go through the 3D dataset slice by slice for selecting the most accurate contours from which the relevant regions or volumes are carefully generated [14]. However, if the person drawing the contours is not a medical expert or radiologist, it will most likely generate poor segmentation results. The task of delineating the fat pads slice by slice sometimes limits the expert’s view and control, and generates jaggy regions. Needless to say, manual fat pads segmentation is also operator dependent and the selected volume is subject to large intra- observer variability.

To evaluate the performance of segmentation algorithm quantitatively, we employed three quality metrics based on voxel ratios: Dice coefficient (DICE), true positive volume fraction (TPVF), and false positive volume fraction (FPVF) [15]. Let *M*_*auto*_ and *M*_*man*_ represent the binary masks produced by the algorithm and the manual segmentation, respectively. Then DICE, TPVF, and FPVF are defined as follows:

$$ DICE=\frac{2\cdot|M_{man}\cap M_{auto}|}{|M_{man}|+|M_{auto}|} $$

$$ TPVF=\frac{|M_{man}\cap M_{auto}|}{|M_{man}|} $$

$$ FPVF=\frac{|M_{auto} \setminus M_{man}|}{|M_{man}|} $$

Segmentation algorithms can be categorized into region-based, boundary-based, or model-based approaches. Region-based approaches may be categorized as being based on thresholding, clustering, or region growing. They do not work very well for inhomogeneous data sets. Boundary-based approaches only work in case of clearly extractable boundaries, while model-based approaches only work if the shape of the to-be-segmented object is well-defined or if a large training dataset in the form of an atlas is available. As, to our knowledge, no existing approaches exist that specifically target fat pad segmentation from MR images, we evaluated our method by comparing against commonly used, generally applicable segmentation algorithms. Since fat pads are not well-defined objects and we do not have large training datasets, we can only compare against region-based and boundary-based approaches. Prominent representatives of the three groups of region-based approaches are multiOtsu thresholding [16], fuzzy c-means clustering [17], seeded region growing [18]. Worth mentioning boundary-based segmentation algorithms are watershed transform [19], and level sets segmentation [20].

The inter-observer reliability [21] is computed in terms of kappa (*κ*) statistics for the manually segmented ground truths. The kappa statistic (*κ*) is a better measure that takes account of the agreement expected solely on the basis of chance.

$$ \text{Kappa }(\kappa) =\frac{O-E}{1-E} $$

where *O* is the observed agreement and *E* is the expected agreement. The kappa statistic indicates how much the actual agreement beyond chance (*O*−*E*) represents relative to this potential (1−*E*). We compared our results against manually established ground truths. Our results lie within the range of inter-observer variability.

### Material

The Study of Health in Pomerania (SHIP) [22], a cohort study conducted in Northeast Germany, provides us the test datasets. In the SHIP study, more than 3 400 participants aged 20 to 89 years participated. The test datasets are isotropic, three dimensional T1-weighted head MRI with an image size of 176×256×176 voxels, where each voxel has an isotropic volume of 1 *mm* ^3^. These head MR images contain only the upper airways and its surrounding tissues which are considered to contain the most essential information to study the OSA syndrome in the frame of an epidemiological study. Upper airway imaging is a best way to examine the pharyngeal airway and its surrounding soft tissues. Magnetic resonance scanning is considered an ideal modality for soft tissues, since it can accurately quantify pharyngeal fat pads in axial, sagittal and coronal planes without radiation [23].

Thirty individual head MRI datasets were randomly selected for our experiments and tests. Among the 30 datasets, 10 were selected randomly for generating ground truths by manual segmentation by two observers under the supervision of an experienced radiologist.

### Para-pharyngeal fat pads segmentation

**Fig. 2:**
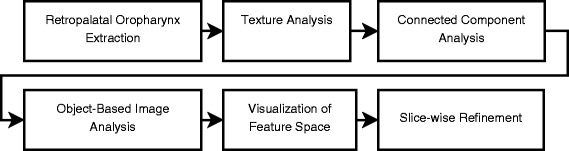
Complete segmentation pipeline

To develop the segmentation algorithm for the fat pads around the oropharynx, we took a number of steps. First, we applied the segmentation algorithm proposed by Shahid et al. [10] to extract the retropalatal oropharynx from MRI datasets. Next, we applied texture analysis to discard the low contrast axial slices in the process of fat pads seed selection. Then, we applied thresholding and connected component analysis to identify 3D objects as initial fat pad candidates. After that, object-based image analysis is employed to extract the features describing the appearance and orientation of each 3D fat pad candidate. These features form a multidimensional feature space, which is visually explored by an interactive visual analysis tool to distinguish fat pad objects from other candidate objects. Then, a supervised classifier is designed to select the fat pad objects. Finally, fat pad objects are refined and redundant voxels or regions are discarded in a slice-wise refinement step. The complete segmentation pipeline of our algorithm is shown in Fig. 2. Our approach does not need any user interaction and is fully automatic. We evaluated our results on ten datasets using a manually segmented ground truth. The following subsections describe each step of the pipeline in detail.

### Retropalatal oropharynx extraction

**Fig. 3:**
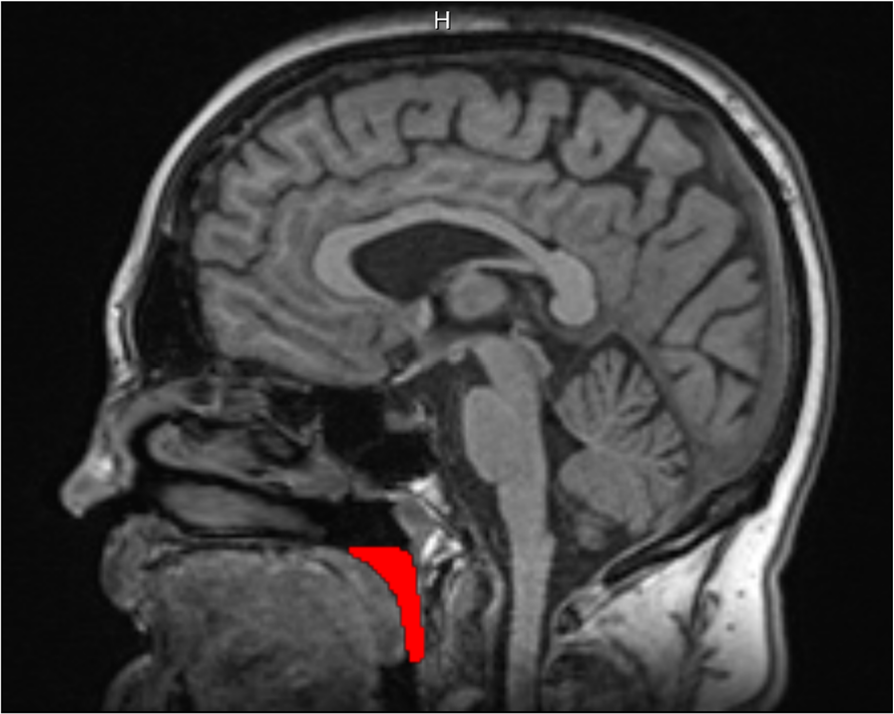
*Red color* represents the retropalatal region of oropharynx

For the analysis of obstructive sleep apnea syndrome, we are interested in the analysis and segmentation of the fat pads around the retropalatal oropharynx which is considered to be the narrowest region of the upper airway [10]. In Fig. 3, red color is used to represent the retropalatal oropharynx. However, all MRI datasets do not start at identical position with respect to the body of the patient. The oropharynx regions can start at different axial slices in the head MRI datasets of our subjects. Therefore, we extracted the retropalatal oropharynx region automatically using the segmentation algorithm developed by Shahid et al. [10].

### Texture analysis

Texture analysis describes the characteristics of image regions by their texture content. Texture analysis can be useful when a region in an image is more characterized by its texture than by gray levels. We attempt to quantify the intuitive qualities such as smooth, homogeneous, or random as a function of the intensity variations. Homogeneous regions have no variation, smooth regions are slowly changing and as such have small local variations, while random parts exhibit largest variations. Hence, standard statistical measures (standard deviation, entropy, homogeneity, average, variance etc.) are used to characterize the texture of a region in our image. They indicate the local variability of voxel gray levels in the image.

We applied texture analysis to discard the axial slices having relatively smooth regions and low contrast in the process of obtaining initial 3D objects for fat pads. In low contrast axial slices, we have region leakage problem for the fat pads which causes strong artifacts in the shape of fat pads. These artifacts lead to uncertain variations in the shape features of the fat pad candidates. Omission of the low contrast axial slices during the seed selection process does not have any major effect on the segmentation process because we just need a good starting axial slice as a starting point for our segmentation algorithm. In this way, we achieve more consistency in terms of shape features.

### Connected component analysis

After having reduced the number of slices to be analyzed in the texture analysis, a median filter of kernel size 3×3×5 is applied to minimize the salt and pepper noise [24] from the head MRI. Then, an anisotropic diffusion filter [25] is applied to smooth the image while keeping the object boundaries intact.

**Fig. 4:**
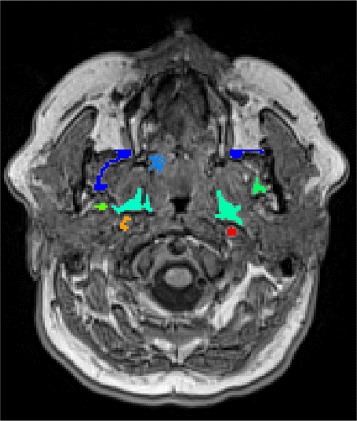
Fat pad candidates on axial slice

There is a number of general segmentation techniques available to generate a set of fat pad candidates by over-segmentation. We apply a coarse intensity clustering [26] to the image volume to generate fat pad candidates by over-segmentation due to its low computational complexity. Since fat pads are bright regions in T1-weighted MRI, we select bright regions in image as fat pad candidates. Figure 4 demonstrates an axial slice of the T1-weighted head MRI, where the bright objects as fat pad candidates are highlighted. After slice-wise intensity clustering, we have bright regions on axial slices of the head MRI datasets which includes the para-pharyngeal fat pads and other bright regions like mandible tissues, and blood vessels.

Having bright regions on axial slices, we connect them using connected component analysis in order to build 3D fat pad candidates. We use a 26-neighborhood relationship in a 3D connected component analysis. As a result to the connected component analysis on bright regions, we obtain a considerably small number of 3D regions that serve as fat pad candidates.

### Object-based image analysis

State-of-the-art segmentation algorithms mainly rely on a voxel level to draw the contours of organs. However, when operating exclusively on a voxel level, only a limited number of features and properties can be utilized to separate the fat pads from surrounding structures and tissues. Thus, we overcome the limitations of voxel-based processing by examining the image as atomic regions (called *objects*) in their local semantic context [27]. Atomic regions introduce additional features that are not available on a voxel level such as intensity statistics, orientation vectors, and shape descriptors.

Within our work, we define the image regions as possible candidates and extract the descriptive features of objects using the methodology of object-based image analysis introduced by Homeyer et al. [27]. They assign to objects a set of features regarding their shape, orientation profile, and intensity statistics. We define features that we consider potentially helpful in describing fat pads and discriminating them from other candidates. Hence, we define features for 3D objects that describe their intensity profile, their relative and absolute position, and their shape.

To describe the intensity profile, we compute different statistical measures of intensity, including minimum, maximum, and average values as well as median, and upper and lower quartiles.

To describe the absolute position of the objects, we use their center of gravity. For the relative position, we compute x-axis and y-axis projections of distances between centers of gravity of pharynx and the fat pad candidates as distance features. This requires us to know the pharynx location. As mentioned above, the pharynx segmentation is obtained using the approach of Shahid et al. [10]. The relative distances are then computed by

$$ X\text{-}axis\_ {Proj.}={(\overline{x}_{obj}-\overline{x}_{ph})}^{2} $$

$$ Y\text{-}axis\_ {Proj.}={(\overline{y}_{obj}-\overline{y}_{ph})}^{2} $$

where $(\overline {x}_{obj},\overline {y}_{obj})$ represents the coordinates of the center of gravity of fat pad objects and $(\overline {x}_{ph},\overline {y}_{ph})$ defines the coordinates of the center of gravity of pharynx.

Many shape descriptors are available in literature including a number of methods to compute the rotation invariant shape features [28]. We apply the concept of central image moments [29] to a 3D setting to compute the shape features of the binary mask of a region *b*, where *b*(*x,y,z*)=1 if the voxel (*x,y,z*) belongs to the region and *b*(*x,y,z*)=0 otherwise. The central image moments of the order *p,q,r* for a binary image *b*(*x,y,z*) are defined by

$$ \mu_{pqr}(b)=\sum_{x}{\sum_{y}{\sum_{z}{{(x-\overline{x})}^{p}{(y-\overline{y})}^{q}{(z-\overline{z})}^{r}{b(x,y, z)}}}} $$

where $(\overline {x},\overline {y}, \overline {z})$ defines the coordinates of the center of gravity and the zero-order moment describes the volume of the region. Furthermore, additional orientation and shape features can be computed by applying the principal component analysis [30] on the voxel distribution of the region. The principal eigenvectors and corresponding eigenvalues (*λ*_1_≥*λ*_2_≥*λ*_3_) are computed by the covariance matrix of *b*, which is defined by

$$cov(b)= \left[ {\begin{array}{ccc} \mu_{200}(b) &\mu_{110}(b) &\mu_{101}(b)\\ \mu_{110}(b) &\mu_{020}(b) &\mu_{011}(b)\\ \mu_{101}(b) &\mu_{011}(b) &\mu_{002}(b)\\ \end{array}} \right]. $$

The principal eigenvectors represent the region’s orientation, while the ratio of their corresponding eigenvalues measures the region’s eccentricity.

$$ eccentricity= 1- \frac{27 \cdot \lambda_{1} \cdot \lambda_{2} \cdot \lambda_{3}} {(\lambda_{1} + \lambda_{2} + \lambda_{3})^{3}}. $$

By using the eigenvalues and vectors, additional orientation features measuring compactness can be derived as

$$ elongation = 1-\sqrt{\frac{\lambda_{1}}{\lambda_{2}}}. $$

Perfect spherical objects have zero elongation. Another feature, *flatness*, is defined as

$$ flatness = 1-\sqrt{\frac{\lambda_{2}}{\lambda_{3}}}. $$

Sheet-like objects have a flatness close to one (for infinitely wide, infinitely thin sheet). Wire-like structures have flatness close to zero, just like spherical objects.

All these descriptive features form a multidimensional feature space, where each object corresponds to a multi-dimensional point in that feature space. We normalized the numerical values of the features to have a uniform range. Now, the task is to explore the multidimensional feature space of useful features in order to determine the decisive contribution of features.

### Visualization of feature space

**Fig. 5:**
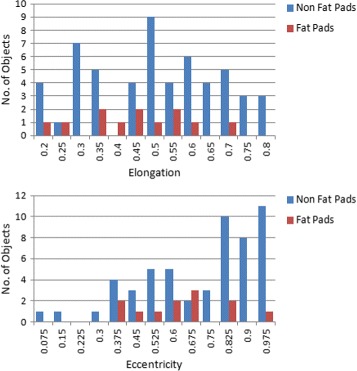
Histograms of individual features. *Top*: Elongation values of 3D objects. *Bottom*: Eccentricity values of 3D objects

We analyze the influence of the features individually using histograms to find a single feature which can serve our purpose in a best way. However, it is evident from their histograms shown in Fig. 5 that there does not exist a single decisive feature which can discriminate fat pad objects from other candidates. Nevertheless, some features have a narrow spread of values with low variance for fat pad objects and their combination with other features might be helpful to single out fat pad objects.

Hence, we visualize the descriptive features collectively, which form a multidimensional feature space, to find suitable weights of feature values for separating our candidates. Dimensionality reduction method is utilized to map multidimensional data to a low-dimensional 2D space for a visual exploration of the data distribution. Principal component analysis (PCA) [30] or multi-dimensional scaling [31] are the most commonly used dimensionality reduction methods. The selection of an optimal method generally depends upon preservation of distances, computational efficiency, and cluster preservation [32]. Since we aim to develop an intuitive interactive system, linear projection is the best available choice, as they are economical in terms of computational cost and do not introduce non-linear distortion, which makes them intuitive.

The linear projection of the n-dimensional feature space *Q* onto a 2-dimensional projection space *U* can be implemented by using a 2×*n* projection matrix *P*. The columns of the projection matrix represent the coordinates of the basis vector images mapped from *Q* to *U* space. Since the origin of the projection space coincides with the origin of feature space, the columns of linear projection matrix are considered as axes of a star-coordinates widget.

**Fig. 6:**
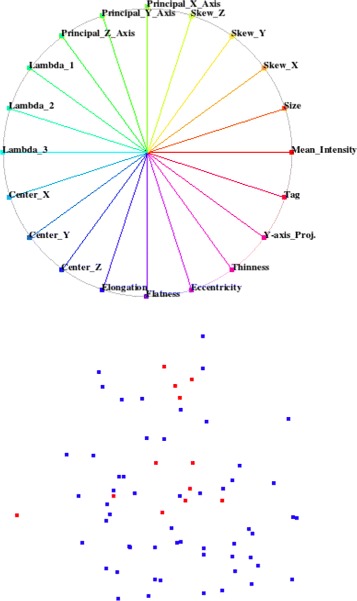
*Top*: Default configuration of star-coordinate widget. *Bottom*: Projected space of default star-coordinates widget

In the default configuration, all axes of the widget are uniformly distributed over the unit circle as shown in Fig. 6, having the linear projection matrix:

$$P= \left[ {\begin{array}{cccc} 1 &\cos{(2\pi/n)} &... &\cos{(2\pi(n-1)/n)}\\ 0 &\sin{(2\pi/n)} &... &\sin{(2\pi(n-1)/n)}\\ \end{array}} \right]. $$

Our interactive visual analysis system allows us to manipulate the projection by varying the positions of the end-points of basis vectors. The projection matrix is then recomputed to project the feature space. Moving the end-point of a basis vector to the origin cancels out the effect of selected feature by leading a corresponding column of zero elements in the projection matrix.

We visualize the training datasets, where we know the class of each sample, to find a configuration of the star-coordinates widget which can visually decouple the fat pads class from the background class. We started with a default configuration of the star-coordinates widget where the uniformly distributed axes of the widget represent the all possible features. We use two colors for the projected samples as shown in Fig. 6, where each color corresponds to a different class. We determine the influence of each individual feature on projected space by varying the length and angle of its corresponding basis vector in the star-coordinates widget. We eliminate the feature from the default configuration of the widget if it affects both classes in a same way and does not help us to visually decouple both classes. In this way, we are left with few efficacious features which distinguish both classes vividly.

**Fig. 7:**
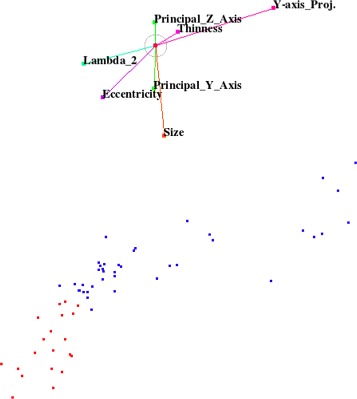
*Top*: Star-coordinate widget configuration. *Bottom*: *Red and blue* samples represent fat pad and non fat pad objects, respectively, in projected view

In general, it is not always possible to find a configuration of the widget that classifies the samples perfectly. However, we achieved a perfect separation of the classes. We found a configuration of the star-coordinates widget that exhibits a region with all red samples representing fat pads in the projected space. There is no blue sample of the background class in that region. We observed that only few salient features suffice to create this projected space as shown in Fig. 7.

After this training phase, we can apply the derived knowledge to any test data or data set with no known ground truth. We saved the respective projection matrix from the training session for the classification of test data with no known ground truth. In order to classify the test data, we map the new feature space using the recorded projection matrix. Then, silhouette coefficients for each sample of the test data when tentatively adding that sample to the cluster of fat pad objects are computed. The best silhouette coefficient, then, selects the sample that is matching the cluster of fat pad objects best, i.e., it is supposed to represent the fat pad in the test data. The silhouette coefficient [33] is defined by

$$ s(i)=\frac{b(i)-a(i)}{\max{\{a(i),b(i)\}}} $$

where *s*(*i*) is the value of the silhouette coefficient for the *i*th sample of the test data, *a*(*i*) is the average of the Euclidean distances from *i*th sample of the test data to all fat-pad samples in the training data, and *b*(*i*) is the average of the Euclidean distances from the *i*th sample of the test data to all non-fat-pad samples in the training data. All fat-pad and non-fat-pad samples are shown as red and blue points, respectively, in the projected space of Fig. 7.

On average, for our data as described above there are twelve samples in each dataset while only two samples are classified as (left and right) fat pads. To evaluate the performance of our classifier in practice, holdout validation method is employed for the thirty datasets. We partitioned our datasets into two sets of ten datasets for training and twenty datasets for testing. We built the classifier using the training set and evaluated it using the testing set. We run multiple rounds of handout validation, and the validation results are averaged over the rounds to reduce the variability. We achieved excellent results for our supervised fat pad classifier which detected all fat pad samples accurately in our datasets with no false positives and no false negatives. The perfectly designed classifier laid the foundation for the complete automation of our segmentation algorithm.

### Slice-wise refinement

Our classified fat pad objects include some false surrounding voxels due to intensity inhomogeneity and low contrast of MR images. More precisely, the connected component step leaked into surrounding regions, which we need to cut off in this last step. We used only intensity clustering for generating the fat pad candidates in the connected component analysis step to keep the algorithm simple and fast which created extra artifacts. Therefore, we need to remove these redundant voxels and regions from fat pad objects in a refinement step. This refinement is performed on a slice-wise basis in axial direction.

We start refining the fat pad object from the axial slice where fat pads tend to be convex. From our a priori domain knowledge, fat pads have close-to-convex shape in general. After finding the best axial slice, we measure the statistics (center of gravity, area, average intensity, and intensity variance) of the fat pad object for processing in other axial slices. First, we move in the downward and then in the upward direction to process all axial slices of fat pad objects. For each axial slice, a specific area around the center of gravity of the adjacent processed axial slice is selected which is still comparatively much smaller than the entire axial slice. Then iterative MultiOtsu thresholding [16] is applied to separate fat pads for statistical analysis. In statistical analysis, different features (center of gravity, area, average intensity, and intensity variance) of delineated regions are computed, and regions like blood vessels and mandible tissues are discarded depending upon a defined criterion. Only those regions are considered as fat pad regions which do not show a variation of more than 30% from the adjacent slice features. It helps us to remove blood vessels (more bright in intensity) and mandible tissues (far away from the center of fat pad) from fat pad objects.

**Fig. 8:**
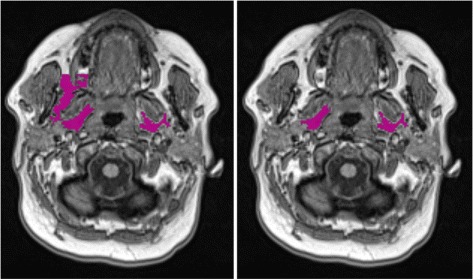
*Left*: Surrounding regions connecting with fat pad. *Right*: Exclusion of surrounding regions

Moreover, we face a problem of leaking regions in few axial slices where regions of fat pad and mandible tissues are connecting with each other as shown in Fig. 8. To address this issue, we build and invert a distance map and then apply a watershed transform [34] to the inverted distance map. The watershed transform separates the connecting regions by building boundaries. After having different regions as a result of the watershed transform, only overlapping regions with the adjacent slice are considered as fat pad regions.

After applying all these refinement steps and procedures, we are left with fat pad objects where all redundant and extra regions have been excluded. In the end, we perform a morphological closing operation [35] to fill the holes and cavities and to remove the boundary irregularities.

## Results

**Fig. 9:**
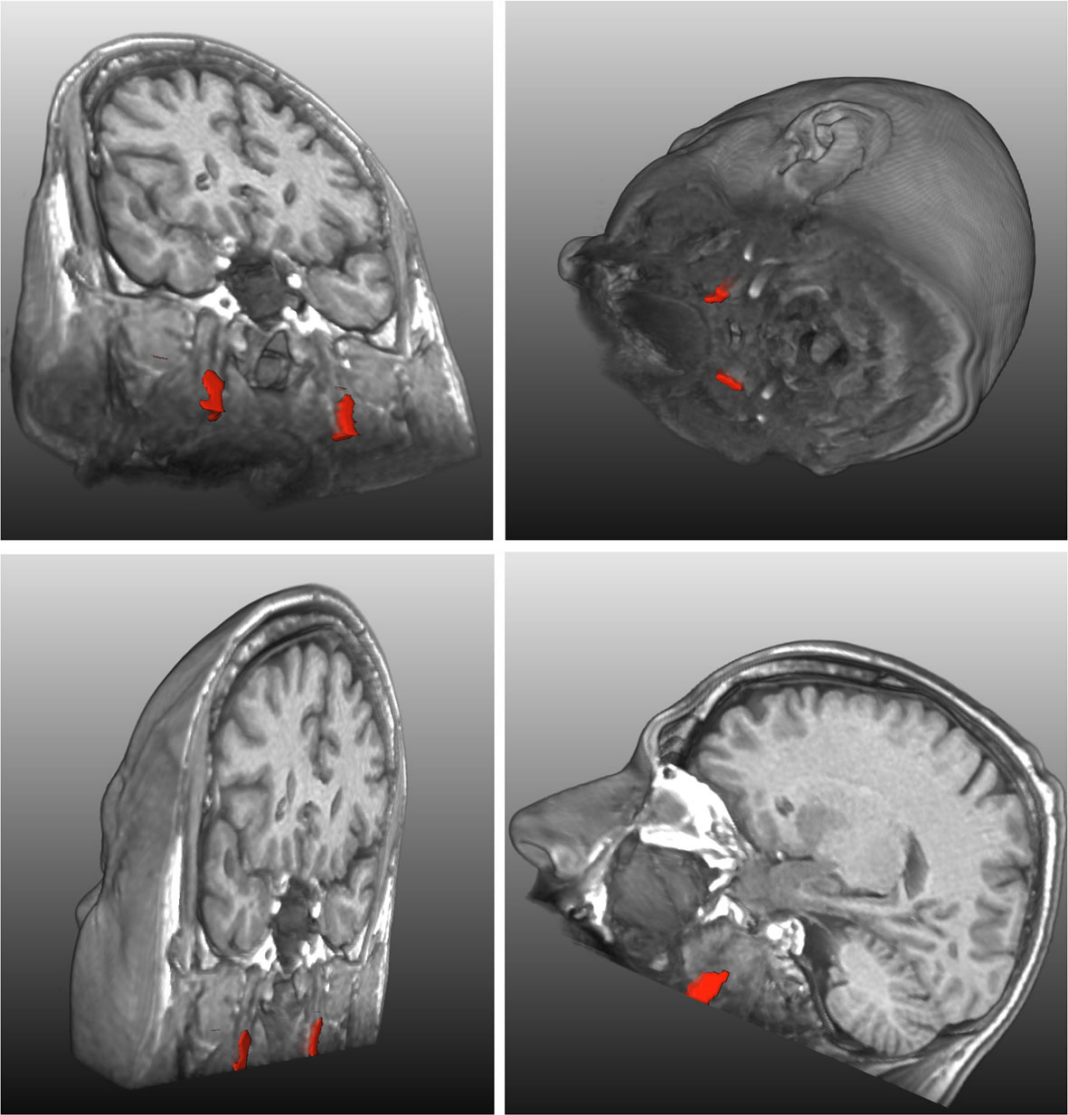
Different views of segmented fat pads

The para-pharyngeal segmentation algorithm has been tested on randomly selected thirty datasets; each dataset represents a separate subject. The processing of each MRI with 176×256×176 voxels takes not more than one minute on a computer with Intel Core i5 2.67 GHz CPU with 4 GB RAM. There is no need to tune any parameter to segregate fat pads from head T1-weighted MR images. To evaluate the accuracy of our supervised classifier, we applied our classifier on thirty datasets. Our supervised classifier produced accurate results in the selection of fat pad candidate from initial 3D objects. We got 100% true positives with 0% false positives. The main reason of such a high positive rate is our pre-processing and working on 3D objects instead of 2D objects. The highly accurate classifier laid the foundation of full automation of the segmentation algorithm. We also analyzed qualitatively the results of our segmentation pipeline on all thirty datasets and we found them promising. The results of our segmentation pipeline on a single subject are shown visually as red regions in Fig. 9.

To compute the quality metrics, we got the manually segmented ground truths for the randomly selected ten datasets from two observers under the supervision of a radiologist. These three metrics are used to compute the results of our segmentation pipeline and to compare them with the ground truths of the second observer against the first observer. We presented our results against the ground truths of the first observer in Table 1. These quality metrics give us a complete insight of our algorithm. They do not only indicate the error but also inform us about the nature and origin of the error. These quality factors show intuitively whether an error is due to wrong inclusion of surrounding voxels or wrong exclusion of object regions. Higher values of DICE and TPVF fraction and lower value of FPVF indicate better results. For ideal results, we would have DICE and TPVF fractions showing 100% results and FPVF fraction indicating 0%.

**Table 1:** Evaluation and comparison of our segmentation results with observer2 against observer1

Masks		DICE	TPVF	FPVF
		(%)	(%)	(%)
Observer2	Avg.	80.4	82.5	22.8
	Std.	3.0	6.5	10.9
Automatic	Avg.	77.9	79.1	24.1
	Std.	4.1	6.8	10.5

**Fig. 10:**
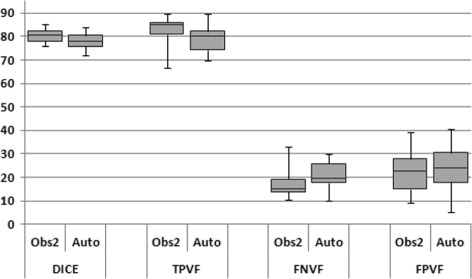
*Box* and whisker plot to compare our segmentation results with manual segmentation of observer2 against observer1

We also visually analyzed our segmentation results in a box and whisker plot in Fig. 10. Table 1 shows that our algorithm performs very well in terms of DICE (mean value is around 78%) and TPVF (mean value is close to 79%). However, the value for FPVF is about 24%. Our results are very close to those of the observer2 in terms of these three metrics against the first observer. We computed *p*-*value* (0.14) for our results and found that the difference between our algorithm and the observer2 is not statistically significant for a significance level of 0.05. The error in our results can be partially explained by the error introduced due to the manual segmentation of ground truth. The manual segmented ground truth is also operator dependent and is subject to large intra- and inter- observer variability. The manual extraction of the fat pads does not produce significantly more accurate results than our segmentation pipeline. The manual error is mainly due to the voxels on the boundary of fat pads which are affected by the partial volume effect. The ambiguous boundary of fat pad emphasizes the subjectivity of the human experts. On each axial slice of our dataset, we have on average 80 voxels for each fat pad and 35 out of them are boundary voxels. As mentioned above, we obtained additional manual segmented ground truths from a second observer to measure inter-observer variability and to understand the amount and the nature of the error better. Furthermore, the head MRI dataset contains only a small volume of fat pad: on average 1 000 to 1 500 voxels of each (left or right) fat pad, so minute deviation or wrong inclusion or exclusion of 20 to 30 voxels per slice produces substantial false positive or false negative results. In addition, we have strong artifacts due to low contrast and inhomogeneity of head MRI datasets which may introduce high false positive and negative errors.

We also measured the inter-observer reliability [21] in terms of kappa (*κ*) statistics for our two manually segmented ground truths. First, we calculated observed agreement (*O*) equal to 0.98 for our ground truths. The observed agreement has the disadvantage that some agreement would exist even if both observers simply guessed the result. We found *κ*=0.8 in our case which indicates a substantial agreement according to the guidelines of Landis and Koch [36].

**Fig. 11:**
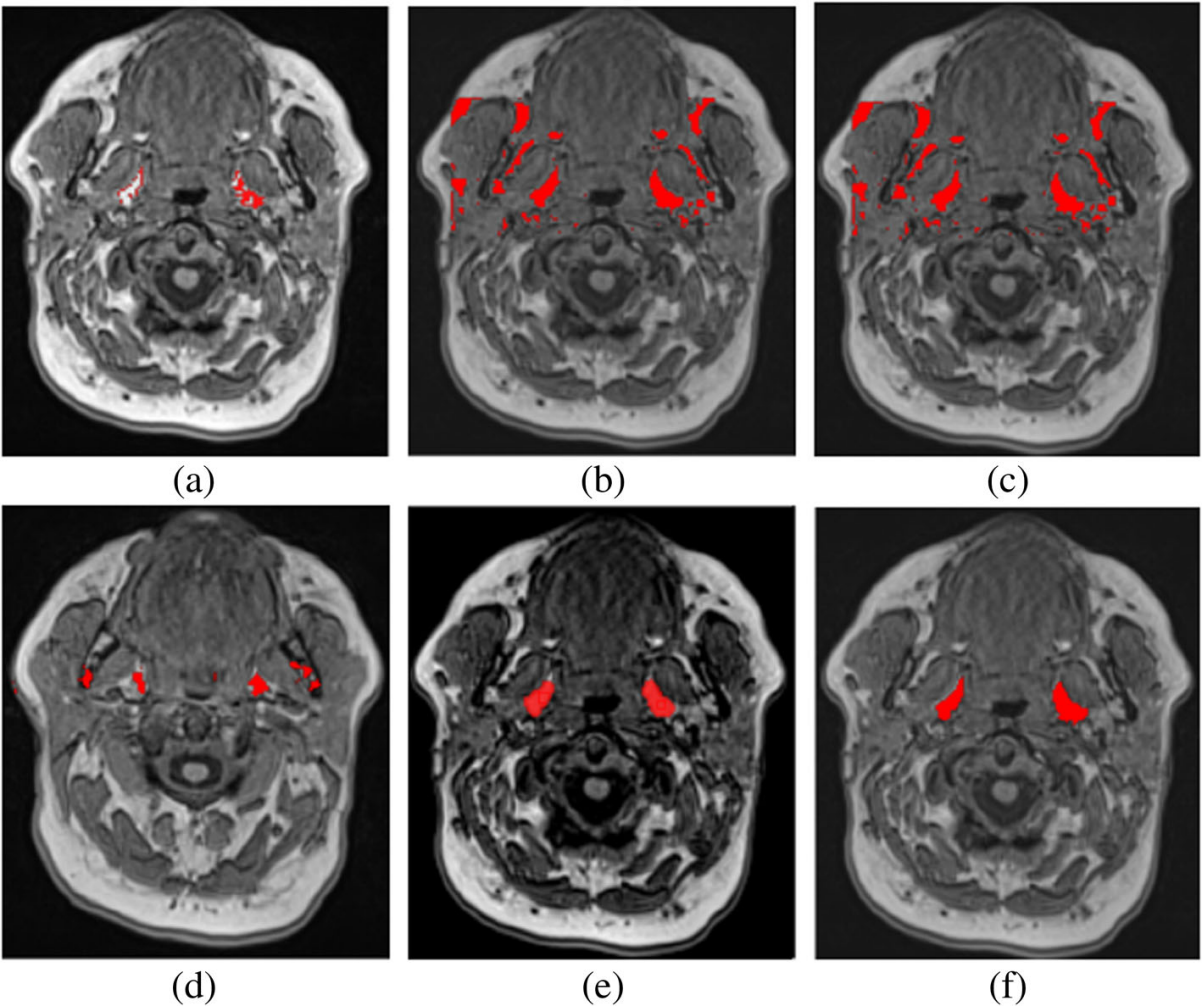
Comparison of different segmentation techniques. Results of fat pads segmentation are shown in *red* color. **a** region growing **b** fuzzy c-means **c** multiOtsu thresholding **d** watershed transformation **e** level sets **f** our algorithm

We compare our results against the five approaches, see Fig. 11. Table 2 summarizes the comparison of our segmentation pipeline against the general segmentation techniques. The generally applicable methods did not produce good results, as they are often embedded in a pipeline with several pre- and post-processing steps.

**Table 2:** Comparison of our segmentation results with generally available segmentation methods

Masks		DICE	TPVF	FPVF
		(%)	(%)	(%)
Our Algorithm	Avg.	77.9	79.1	24.1
	Std.	4.1	6.8	10.5
Region Growing	Avg.	35.8	46.9	181.8
	Std.	18.3	27.3	289.3
Fuzzy c-means	Avg.	43.4	52.3	92.1
	Std.	13.3	20.3	78.4
MultiOtsu	Avg.	39.8	71.6	226.4
	Std.	12.4	10.6	151.5
Watersheds	Avg.	28.6	19.8	10.8
	Std.	23.7	18.3	14.1
Level Sets	Avg.	40.0	43.8	68.3
	Std.	20.6	31.8	119.0

It can be seen that no single algorithm manages to achieve accurate results. Seeded region growing algorithm and level sets segmentation method need user interaction to select the seed points for the segmentation of the object. The seeded region growing method faces severe region leakage problem in low contrast slices of the data set. MultiOtsu thresholding and fuzzy c-means algorithm could not produce better results when applied on complete image. Moreover, multiOtsu thresholding is expensive in terms of time. The watershed transform also faces limitations like over-segmentation and under-segmentation for different slices of a dataset, and could not produce sufficient results. For all the algorithms, preprocessing is applied on the image before the application of the segmentation method. The region growing algorithm faces the problem of region leakage due to the low contrast of the data sets. Other algorithms fail due to the inhomogeneous nature of fat pads. As a comparison to them, our proposed method produces desirable results, does not involve any user interaction and takes less than one minute for each subject.

## Discussion

A fast and fully automatic segmentation technique has been presented to segregate para-pharyngeal fat pads from T1-weighted head MR images. The complete pipeline includes texture analysis, connected component analysis, object-based image analysis, and supervised classifier using interactive visual analysis tool. The algorithm is fast, as the whole processing consumes less than a minute for a single MRI dataset. The method has been tested on thirty random datasets. The proposed algorithm generates sufficiently reliable and accurate results and has potential to be applied for the study of large data in epidemiological studies such as SHIP to understand the pathogenesis of the obstructive sleep apnea syndrome. Our algorithm could also be readily integrated in clinical routine.

Our idea of defining a supervised classifier using the interactive visual analysis tool can be employed to develop a classifier for the segmentation of other organs and tissues. The core idea of our segmentation algorithm can be applied to solve many medical segmentation problems. Initially generating coarse objects as fat pad candidates using intensity clustering improves the speed of our algorithm and then, refining the fat pad object increases the accuracy of our segmentation method.

As future work, we plan to extract different features and properties of segmented fat pads and visualize them to understand the pathogenesis of OSA. Furthermore, we are also interested in studying the role of fat pads in the treatment of OSA.

## Conclusions

The proposed segmentation algorithm delineates para-pharyngeal fat pads automatically without any user interaction. The algorithm is fast enough to be applied in a cohort study to investigate OSA. Different image features like fat pads’ volume, and distance between left and right fat pads are measured from the segmented fat pads. These image features will be further analyzed to determine their associations with OSA.

## Abbreviations

Avg.: Average
CT: Computed tomography
Dist.: Distance
FPVF: False positive volume fraction
MRI: Magnetic resonance imaging
OBIA: Object-based image analysis
OSA: Obstructive sleep apnea
PCA: Principal component analysis
Ph.: Pharynx
Proj.: Projection
Std.: Standard deviation
SHIP: Study of health in Pomerania
TPVF: True positive volume fraction

## References

[1] Sutherland K, Lee RWW, Phillips CL, Dungan G, Yee BJ, Magnussen JS, Grunstein RR, Cistulli PA. Effect of weight loss on upper airway size and facial fat in men with obstructive sleep apnoea. 2011; 66(9):797–803. doi:10.1136/thx.2010.151613. 10.1136/thx.2010.151613 21680567

[2] Pack AI (2002). Sleep Apnea: Pathogenesis, Diagnosis and Treatment.

[3] Lowe AA, Fleetham JA. Two- and three-dimensional analyses of tongue, airway, and soft palate size. Atlas of the Difficult Airway In: Norton ML, Brown ACD, editors. Mosby-Year Book, St. Louis: 1991. p. 74–82.

[4] Young T, Peppard PE, Taheri S (2005). Excess weight and sleep-disordered breathing. J Appl Physiol.

[5] Grunstein RR, Stenlöf K, Hedner JA, Peltonen M, Karason K, Sjöström L (2007). Two year reduction in sleep apnea symptoms and associated diabetes incidence after weight loss in severe obesity. Sleep.

[6] Schwartz AR, Patil SP, Laffan AM, Polotsky V, Schneider H, Smith PL (2008). Obesity and obstructive sleep apnea: pathogenic mechanisms and therapeutic approaches. Proc Am Thorac Soc.

[7] Schwab RJ, Pasirstein M, Pierson R, Mackley A, Hachadoorian R, Arens R, Maislin G, Pack AI (2003). Identification of upper airway anatomic risk factors for obstructive sleep apnea with volumetric magnetic resonance imaging. Am J Respir Crit Care Med.

[8] Watanabe T, Isono S, Tanaka A, Tanzawa H, Nishino T (2002). Contribution of body habitus and craniofacial characteristics to segmental closing pressures of the passive pharynx in patients with sleep-disordered breathing. Am J Respir Crit Care Med.

[9] Ivanovska T, Dober J, Laqua R, Hegenscheid K, Völzke H, Bebis G (2013). Pharynx segmentation from MRI data for analysis of sleep related disoders. Advances in Visual Computing.

[10] Shahid MLUR, Chitiboi T, Ivanovska T, Molchanov V, Völzke H, Hahn HK, Linsen L. Automatic pharynx segmentation from MRI data for obstructive sleep apnea analysis. In: Proceedings of the 10th International Conference on Computer Vision Theory and Applications: 2015. p. 599–608. doi:10.5220/0005315905990608.

[11] Ivanovska T, Laqua R, Shahid ML, Linsen L, Hegenscheid K, Völzke H (2015). Automatic pharynx segmentation from MRI data for analysis of sleep related disorders. Int J Artif Intell Tools.

[12] Liu J, Udupa JK, Odhnera D, McDonough JM, Arens R (2003). System for upper airway segmentation and measurement with mr imaging and fuzzy connectedness. Acad Radiol.

[13] Ivanovska T, Buttke E, Laqua R, Völzke H, Beule A. Automatic trachea segmentation and evaluation from MRI data using intensity pre-clustering and graph cuts. In: Image and Signal Processing and Analysis (ISPA), 2011 7th International Symposium On. Dubrovnik: 2011. p. 513–8. IEEE.

[14] Gordillo N, Montseny E, Sobrevilla P (2013). State of the art survey on MRI brain tumor segmentation. Magn Reson Imaging.

[15] Udupa JK, LaBlanc VR, Schmidt H, Imielinska C, Saha PK, Grevera GJ, Zhuge Y, Currie L, Molholt P, Jin Y (2002). Methodology for evaluating image-segmentation algorithms. Medical Imaging 2002.

[16] Liao PS, Chen TS, Chung PC (2001). A fast algorithm for multilevel thresholding. J Inf Sci Eng.

[17] Bezdek JC, Ehrlich R, Full W (1984). Fcm: The fuzzy c-means clustering algorithm. Comput Geosci.

[18] Adams R, Bischof L (1994). Seeded region growing. IEEE Trans Pattern Anal Mach Intell.

[19] Beucher S, Meyer F (1992). The morphological approach to segmentation: the watershed transformation. Opt Engineering-New York-Marcel Dekker Inc.

[20] Paragios N, Deriche R (2000). Geodesic active contours and level sets for the detection and tracking of moving objects. IEEE Trans Pattern Anal Mach Intell.

[21] Bland JM, Altman DG (1986). Statistical methods for assessing agreement between two methods of clinical measurement. Lancet.

[22] Völzke H, Alte D, Schmidt CO, Radke D, Lorbeer R, Friedrich N, Aumann N, Lau K, Piontek M, Born G, et al. Cohort profile: the study of health in pomerania. Int J Epidemiol. 2011;294–307. 10.1093/ije/dyp394 20167617

[23] Welch KC, Foster GD, Ritter CT, Wadden TA, Arens R, Maislin G, Schwab RJ (2002). A novel volumetric magnetic resonance imaging paradigm to study upper airway anatomy. Sleep, New York.

[24] Gonzalez RC, Woods RE. Digital image processing: Pearson prentice hall. Upper Saddle River, NJ; 2008.

[25] Perona P, Malik J (1990). Scale-space and edge detection using anisotropic diffusion. Pattern Anal Mach Intell IEEE Trans.

[26] Shapiro LG, Stockman GC. Computer Vision. New Jersey, Prentice-Hall; 2001, pp. 279–325. ISBN 0-13-030796-3.

[27] Homeyer A, Schwier M, Hahn HK. A generic concept for object-based image analysis. In: VISAPP (2): 2010. p. 530–3.

[28] Yang M, Kpalma K, Ronsin J, et al. A survey of shape feature extraction techniques. Pattern Recogn. 2008;43–90.

[29] Burger W, Burge MJ (2009). Principles of Digital Image Processing.

[30] Jolliffe I (2005). Principal Component Analysis.

[31] Borg I, Groenen PJ (2005). Modern Multidimensional Scaling: Theory and Applications.

[32] Molchanov V, Chitiboi T, Linsen L. Visual analysis of medical image segmentation feature space for interactive supervised classification. In: Eurographics Workshop on Visual Computing for Biology and Medicine: 2015. p. 11–19. doi:10.2312/vcbm.20151204.

[33] Rousseeuw PJ (1987). Silhouettes: a graphical aid to the interpretation and validation of cluster analysis. J Comput Appl Math.

[34] Vincent L, Soille P (1991). Watersheds in digital spaces: an efficient algorithm based on immersion simulations. IEEE Trans Pattern Anal Mach Intell.

[35] Soille P (1999). Morphological Image Processing: Principles and Applications.

[36] Landis JR, Koch GG (1977). The measurement of observer agreement for categorical data. Biometrics.

